# Comparative proteomics and micro-RNA analysis of skeletal muscle cell small extracellular vesicles - Unique profiles in cells from severely obese individuals with type 2 diabetes *versus* normal glucose tolerance

**DOI:** 10.3389/fphys.2025.1696916

**Published:** 2025-11-19

**Authors:** Stian Forstrøm Christiansen, Kari Bente Foss Haug, Misbah Hussain, Abdille Hussein, Berit Sletbakk Brusletto, Reidun Øvstebø, Oliwia Witczak, Vigdis Aas

**Affiliations:** 1 Department of Life Sciences and Health, Oslo Metropolitan University, Oslo, Norway; 2 Department of Medical Biochemistry, Oslo University Hospital, Oslo, Norway

**Keywords:** extracellular vesicles, skeletal muscle, type 2 diabetes, proteomics, micro-RNA, obesity, myotube

## Abstract

Type 2 diabetes (T2D) is associated with increased morbidity, mortality, and substantial healthcare costs. Peripheral insulin resistance, involving interconnected dysregulation of multiple organs, is considered a major driver of T2D. Extracellular vesicles (EVs) are suggested as mediators of this dysregulation based on their properties in intercellular communication. Given the role of skeletal muscle in glucose metabolism, the content of skeletal muscle-derived EVs may provide insights into mechanisms of T2D. To examine this, myotubes from severely obese female T2D donors and matched women with normal glucose tolerance (NGT) were cultured. Small EVs (sEVs) were isolated by differential centrifugation and filter columns and characterized by nanoparticle tracking analysis, flow cytometry, and transmission electron microscopy. The micro-RNA (miR) content of sEV was analyzed via Affymetrix microarray, while proteins were detected by LC-MS/MS. No group differences were found in sEV concentration, size, or EV-marker levels. In total, 495 proteins were detected in the sEVs, of which 55 were unique to the T2D group and 2 to the NGT group. Principal component analysis showed distinct clustering, demonstrating clearly different protein profiles. Quantification of the protein cargo revealed 194 proteins with significantly higher levels and 21 with significantly lower levels in the T2D group. While 208 miRs were detected, no significant group differences were observed. However, 40 miRs were unique to the T2D group and 5 to the NGT group. Pathway analysis of protein and miR data revealed associations with EV-related mechanisms such as exocytosis and protein homeostasis, as well as T2D-relevant pathways including some involved in glucose metabolism, inositol metabolism, and extracellular matrix organization. In conclusion, myotube-derived sEVs from severely obese female donors with or without T2D showed distinct proteome-profiles, however, no differences were observed in the miR content. Other sEV characteristics were similar between the groups.

## 1 Introduction

Type 2 diabetes (T2D) is characterized by a progressive decline in peripheral insulin sensitivity, leading to metabolic dysregulation and eventual deficits in pancreatic β-cell function and survival. This impairment limits systemic glucose uptake, resulting in sustained hyperglycemia, often accompanied by elevated levels of circulating lipids and pro-inflammatory cytokines (DeFronzo and Tripathy, 2009). T2D significantly increases the risk of cardiovascular disease, cancer, and overall mortality (Baena-Diez et al., 2016). While considerable progress has been made in understanding the pathophysiology of T2D, the underlying mechanisms of the disease are not fully understood. Chronic low-grade inflammation, ectopic fat accumulation, and endoplasmic reticulum stress in adipose tissue, skeletal muscle, and liver are thought to contribute to disease progression (Mastrototaro and Roden, 2021). However, the complex interactions between these tissues need further investigations. In this context, extracellular vesicles (EVs) and their cargo may play a key role in mediating inter-tissue communication in T2D (Castaño et al., 2018).

EVs are nano-sized particles with a lipid bilayer that contain bioactive molecules such as proteins, lipids, and nucleic acids. EVs are commonly categorized by size into large (>200 nm diameter) and small EVs (sEVs, <200 nm diameter), with sEVs often enriched in exosomes (Welsh et al., 2024). Exosomes are of particular interest, as their biosynthesis within the endosomal system involves selective molecular sorting, allowing specific proteins and micro-RNAs (miRs) to be packaged and secreted (Garcia-Martin et al., 2022; Lee et al., 2024). Upon release in all body fluids, EVs can transfer their cargo to recipient cells and thereby alter cellular phenotype and function (Colombo et al., 2014). The circulating EV numbers have been reported to be elevated in both obesity (Amosse et al., 2018) and T2D as summarized in the meta-analysis by Li and coworkers (Li et al., 2016), with positive correlations observed between EV levels and BMI, blood pressure, and biomarkers of insulin resistance and β-cell dysfunction (Kobayashi et al., 2018). Functionally, EVs have been linked to the development of insulin resistance and T2D. For example, adipose tissue-derived EVs can promote inflammation and insulin resistance (Deng et al., 2009; Kranendonk et al., 2014a; Kranendonk et al., 2014b; Yu et al., 2018; Dang et al., 2019; Wang et al., 2025). Furthermore, EVs from obese mice induce insulin resistance when transferred to lean mice, whereas EVs from lean mice improve insulin sensitivity in obese recipients (Ying et al., 2017). Similarly, plasma EVs from obese women have been shown to reduce insulin sensitivity in adipocytes (Mleczko et al., 2018).

While most EV-studies in the context of T2D have been done on adipose tissue and macrophages (Deng et al., 2009; Kranendonk et al., 2014a; Kranendonk et al., 2014b; Ying et al., 2017), skeletal muscle is the primary site of glucose uptake in the body, and insulin resistance in this tissue is considered the primary defect in T2D pathogenesis (DeFronzo and Tripathy, 2009). Although only approximately 5% of circulating EVs are estimated to originate from skeletal muscle (Estrada et al., 2022), they may accumulate in the skeletal muscle interstitial space and can therefore exert significant effects on the skeletal muscle tissue itself (Watanabe et al., 2022). The potential negative impact of skeletal muscle-derived EVs is supported by studies in mice, showing that EVs from skeletal muscle in animals fed with a high-fat diet can affect β-cells, the liver, and other skeletal muscle cells, suggesting that skeletal muscle-derived EVs may be involved in the progression of insulin resistance (Aswad et al., 2014; Jalabert et al., 2016). However, how the cargo of skeletal muscle-derived EVs differs between individuals with T2D and normal glucose tolerance (NGT) remains unknown. This comparison could reveal distinct molecular signatures in skeletal muscle-derived EVs that might contribute to the development of insulin resistance in T2D.

Due to the low abundance of skeletal muscle-derived EVs in circulation and the lack of specific skeletal muscle-EV markers (Estrada et al., 2022), *in vitro* experiments stand out as the best way of investigating this group of vesicles. Cultured human myotubes are known to maintain the characteristics of the donors (Henry et al., 1995), which we previously have shown using cells from the same donors (Bakke et al., 2015; Feng et al., 2015), and can therefore be used to investigate skeletal muscle-derived EVs of T2D donors compared to NGT. In this study we aimed to compare size, number, and the cargo of proteins and miR in sEVs derived from human myotubes of obese donors with T2D and NGT. This comparison could provide valuable insights into the mechanisms of skeletal muscle insulin resistance and T2D progression.

## 2 Material and methods

### 2.1 Study Overview and Ethical Approval

Human skeletal muscle cells from severely obese female donors (BMI ranging from 38–54 kg/m^2^) with and without T2D were grown to myotubes *in vitro* over a 7-day differentiation period. sEVs from the final 24 h of culturing in serum-free media were harvested. The concentration, size, and characteristics of the sEVs were determined and compared between the groups, and the content of proteins and miR was identified and compared.

The study was approved by the Institutional Review Board and Regional Committee for Medical and Health Research Ethics of South-East Norway REK (S-09078d, 2009/166), and the biopsies were obtained after informed written consent. The study adhered to the Declaration of Helsinki.

### 2.2 Culturing of human myotubes

Skeletal muscle cell cultures were established by isolation and proliferation of satellite cells from biopsies samples from *m. obliquus internus abdominis* taken from 12 severely obese female donors during gastric bypass surgery. Of these, 6 donors were diagnosed with T2D, and 6 served as controls with NGT. Diagnosis of T2D was based on fasting plasma glucose >7.0 mmol/L, HbA1c > 6.5%, or the use of at least one antidiabetic medication.

Skeletal muscle biopsies were cleaned of adipose tissue and minced using scalpels, before being enzymatically digested (Yasin et al., 1977) with 0.1% trypsin (Thermo Fisher Scientific, Waltham, USA) in Dulbecco’s modified eagle medium (DMEM; Sigma-Aldrich, Burlington, USA) three times for 20 min. All supernatants were collected, and cells seeded in Skeletal Muscle Cell Growth Medium-2 BulletKit (Lonza, Basel, Switzerland). Fibroblasts were removed by a pre-plating step while splitting the cells, repeated twice before storage in liquid nitrogen (Gaster et al., 2001).

The cells were later grown in T75-flasks pre-coated with extracellular matrix gel (Sigma-Aldrich, Burlington, USA) during the differentiation experiment. For proliferation of myoblasts DMEM (5.5 mM glucose) supplemented with 2% Fetal bovine serum (Thermo Fisher Scientific, Waltham, USA) and 2% Ultroser G (Pall, Cergy-Saint-Christophe, France) was used. The culture medium was changed to DMEM (5.5 mM glucose) containing 2% fetal bovine serum and 25 pM insulin (Novo Nordisk, Bagsvaerd, Denmark) at approximately 80% confluence to initiate differentiation into multinucleated myotubes. Both media contained 50 μg/mL gentamicin (Thermo Fisher Scientific, Waltham, USA) and 1.25 μg/mL amphotericin B (Thermo Fisher Scientific, Waltham, USA). The cells were allowed to differentiate for 7 days, incubated in a humidified CO_2_ (5%) atmosphere at 37 °C, with medium changed every two to 3 days.

### 2.3 Isolation of sEVs

On day 6 of differentiation, cell medium was changed to serum free DMEM (5.5 mM glucose) with 25 pM insulin, gentamicin (50 μg/mL) and amphotericin B (1.25 μg/mL). The cells were incubated for another 24 h for synthesis and secretion of EVs, before media were collected, remnant cells and cell debris were removed by centrifugation at 450 *g* for 5 min, and the final cell free supernatants were stored at −80 °C.

The media underwent an initial centrifugation at 17,000 g for 30 min using a fixed-angle Sorvall SS-34 rotor (Kendro Laboratory Products, Newtown, USA), before the supernatant was filtered through a 0.22 µm filter (Merck, Rahway, USA). The supernatant was transferred to Centricon-70 Plus 100 kDa Centrifugal Filter Columns (Merck, Rahway, USA) and centrifuged at 3 500 g for 15 min. The filters were washed with phosphate buffered saline (PBS) and further centrifuged at 3 500 g for 10 min. Finally, the filters were turned upside down, centrifuged at 1 000 g for 2 min, and the sEV filtrates were collected. All centrifugations were performed at room temperature, and all samples were aliquoted and stored at −80 °C. Serum free media was analyzed in parallel to ensure a low number of contaminating particles.

### 2.4 Characterization of sEVs

sEVs were characterized following MISEV standards (Welsh et al., 2024), using nanoparticle tracking analysis, transmission electron microscopy, and flow cytometry. Additionally, we have previously confirmed the presence of CD63 and HSC70/HSP70 and the absence of calnexin by Western blotting using cells from the same donors and the same isolation method (Aas et al., 2023).

#### 2.4.1 Analysis of sEV size and concentration using nanoparticle tracking analysis

The sEVs concentration and size distribution were determined by nanoparticle tracking analysis, using a NanoSight NS500 (Malvern, Amesbury, United Kingdom). sEV isolates were diluted in PBS (0.02 µm-filtered) to be within the recommended concentration range of 1.0-9.0 × 10^8^ particles/mL. Samples were injected at a constant flow using a syringe pump speed of 20, and three videos of 60 s were captured for each sample using a camera level of 14. Analysis was done using the NTA 3.1 software using a detection level of 4 (Malvern, Amesbury, United Kingdom). This method of vesicle quantification is previously shown to be reliable (Vestad et al., 2017). Total sEV number was further normalized to the protein content in the cells of origin, measured by Bradford assay (Bio-Rad Laboratories, Hercules, USA).

#### 2.4.2 Imaging of sEVs using transmission electron microscopy

Fresh sEV isolates were fixed in 5% paraformaldehyde for 5 min. Next, the isolates were attached to formvar/carbon grids for 20 min at room temperature, before the grids were washed first with PBS, then double distilled water. The sEVs were stained with 0.4% uranyl acetate/1.8% methyl cellulose for 2 min, and excess solution gently removed with filter paper. Finally, the samples were observed with a FEI CM200 transmission electron microscope (Philips, Amsterdam, Netherlands) at 120 kV, and images obtained using a Quemesa CCD digital camera (Olympus Soft Imaging Solutions GmbH, Münster, Germany).

#### 2.4.3 Identification of sEV markers CD63 and CD81 by flow cytometry

Immunoaffinity detection of the EV membrane markers CD63, and CD81 was performed using the Exosome CD81 Flow Detection Kit (ThermoFisher Scientific, Oslo, Norway). A total of 30 µL of the sEV isolate was mixed with 70 µL assay buffer, and incubated with anti-CD81 coated Dynabeads (2.7 mm) overnight at 4 °C. The next day, beads were washed three times with PBS (0.1 µm-filtered) containing 0.1% bovine serum albumin. Subsequently, the beads were incubated for 45 min at room temperature with anti-CD63 and anti-CD81 R-phycoerythrin-conjugated antibodies or isotype control (IgG1-RPE, BD Biosciences, Oslo, Norway), followed by two additional washes. Flow cytometry analysis was performed with a BD Accuri™ C6 Cytometer (BD Biosciences, Oslo, Norway). A total of 3000 single events were measured, and median fluorescence intensity was calculated as the signal to noise ratio relative to isotype.

### 2.5 Assessment of sEV Protein cargo using LC-MS/MS

#### 2.5.1 Protein isolation and quantification

For quantification of sEV proteins, samples were lysed by adding 5x RIPA buffer (Thermo Fisher Scientific, Waltham, USA) containing protease inhibitor cocktail (complete, Mini, EDTA-free Protease Inhibitor Cocktail 25x, Roche, Basel, Switzerland), sonicated for 20 s and further lysed on ice for 15 min. Next, BCA assay (Thermo Fisher Scientific, Waltham, USA) was used to determine total protein concentration.

#### 2.5.2 Liquid Chromatography - Mass spectrometry

sEVs corresponding to 20 µg of protein from the sEV suspension were subjected to proteomic analysis. The vesicles were lysed with ProteaseMAX™ Surfactant (Promega), and proteins were reduced, alkylated and digested into peptides with trypsin (Promega). The resulting peptides were desalted and concentrated before mass spectrometry by the STAGE-TIP method using a C18 resin disk (3M Empore), before analysis by nanoLC-MS/MS using an nEASY-LC coupled to a Q Exactive mass spectrometer (Thermo Fisher Scientific, Bremen, Germany) with EASY Spray PepMap® RSLC column (C18, 2 μL, 100Å, 75 μm × 25 cm) and a 60 min LC separation gradient. Two samples were excluded due to quality issues, resulting in a final sample size of n = 5 per group for the proteomic analysis.

The resulting MS raw files were submitted to the MaxQuant software version 1.6.1.0 for protein identification and label-free quantification (LFQ). Carbamidomethyl (C) was set as a fixed modification and acetyl (protein N-term), carbamyl (N-term) and oxidation (M) were set as variable modifications. First search peptide tolerance of 20 ppm and main search error 4.5 ppm were used. Trypsin without proline restriction enzyme option was used, with two allowed miscleavages. The minimal unique + razor peptides number was set to 1, and the allowed FDR was 0.01 (1%) for peptide and protein identification. Label-free quantitation was employed with default settings. The UniProt database with ‘human’ entries (September 2018) was used for the database searches. Only proteins identified in at least three samples in at least one group were included. The MS intensities were LFQ-normalized, log10 transformed, and missing values were imputed from normal distribution. Differences between groups were assessed using unpaired t-tests and permutation-based false discovery rate correction. Q-values <0.05 were considered statistically significant.

### 2.6 Analysis of sEV micro-RNA content by microarray

#### 2.6.1 RNA isolation and quantification

RNA was isolated from sEV samples using the miRNeasy Micro kit (Qiagen, Hilden, Germany) following the manufacturer’s instructions. Briefly, 100 μL sEV isolate was mixed with QIAzol and spiked with 2 μL ath-miR-159a as a normalization control. Chloroform was added for phase separation, and the aqueous layer was mixed with ethanol and applied to RNeasy MinElute columns. Membranes were washed with buffers RWT, RPE, and 80% ethanol, dried, and RNA was eluted in 14 μL RNase-free water. RNA quantity and quality were assessed by spectrophotometry using a NanoDrop One (Thermo Fisher Scientific, Waltham, USA), and eluates were subsequently stored at −80 °C for later analysis.

#### 2.6.2 miR-transcriptomics

A total of 5 ng RNA was subjected to poly-A tailing, and biotin labelling using FlashTag Biotin HSR RNA Labeling kit (Thermo Fisher Scientific, Waltham, USA) following the manufacturer’s instructions. miR transcriptomics was then performed using Affymetrix GeneChip miRNA 4.0 arrays (Thermo Fisher Scientific, Waltham, USA) following the manufacturer’s instructions. Briefly, following the tailing and biotin-labelling the samples were hybridized for 18 h at 48 °C. The arrays were then washed using the Genechip Fluidics Station 450 (Thermo Fisher Scientific, Waltham, USA), and signal intensities read with the 30007G gene array scanner (Hewlett Packard, Spring, USA). One sample in the NGT group was excluded due to quality issues, resulting in a final sample size of n = 5 for NGT and n = 6 for T2D.

Normalization of signal intensities and quality control were done using the Affymetrix® GeneChip™ Command Console software (Thermo Fisher Scientific, Waltham, USA). The intensities were RMA normalized and log2-transformed. miRs with signal intensity above 5 in at least three samples in one of the groups were included. Differences between groups were investigated in R version 4.4.2 (R Foundation for Statistical Computing, Vienna, Austria) using unpaired t-tests and Benjamini–Hochberg correction of p-values. p-values <0.05 were considered significant.

#### 2.6.3 Confirmation of miRNAs by RT-qPCR

Isolated RNA was converted to cDNA using TaqMan® Advanced miRNA cDNA Synthesis kit (Thermo Fisher Scientific, Waltham, USA), and RT-qPCR was performed using a ViiA™ 7 real-time PCR instrument (Thermo Fisher Scientific, Waltham, USA). The sEV-content of the myo-miRs miR-133a, miR-1-3p, and miR-206 were analyzed, along with miR-23a, one of the miRs showing the greatest difference between groups in the array data. Reactions were performed in singles, with the number of donors per group varying depending on the miR of interest (miR-1-3p: T2D = 6, NGT = 5, miR-133a: T2D = 6, NGT = 5, miR-206: T2D = 6, NGT = 4, miR-23a: T2D = 2, NGT = 2). ath-miR-159a was spiked in as an endogenous control, with an average Ct of 23 and a CV of 0.03. Fold changes were calculated using the ΔΔCt method, relativizing the Ct values to the spike in controls and the mean of the NGT group.

### 2.7 Pathway analysis by Ingenuity Pathway Analysis

Protein and miR pathway connections were analyzed using Ingenuity Pathway Analysis (IPA; Qiagen, Hilden, Germany) to link our findings to known canonical and metabolic pathways. IPA was also used to predict miR targets, considering only experimentally validated interactions or high-confidence predictions from TarBase, TargetScan, miRecords, and Ingenuity Expert Findings. Pathway associations are reported as–log_10_(p-value) to accommodate extremely small values (e.g., p = 0.05 corresponds to–log_10_(p-value) = 1.3). IPA additionally provides z-scores, which estimate the direction and magnitude of pathway regulation (positive = activation, negative = inhibition). Because z-scores rely on quantitative measurements, they cannot be applied to predicted miR target genes.

### 2.8 Statistics

All other statistical analysis were performed using Microsoft Excel or R version 4.4.2. Data are presented as mean ± standard deviation (SD) or as mean ± standard error of the mean (SEM) as stated. Fold change is reported as the ratio of T2D results relative to NGT results.

## 3 Results

### 3.1 Characteristics of human donors with and without T2D

Myotubes were derived from skeletal muscle biopsies of 12 severely obese female donors, of which 6 were diagnosed with T2D, and 6 served as controls with NGT. None of the NGT donors used glucose-regulating medication, however one was using statins (simvastatin). In contrast, all T2D donors received glucose-regulating medication (two metformin alone, one metformin and glimepiride, one insulin Insulatard, and two unspecified), and two were using statins (one simvastatin, one unspecified). The T2D group had higher HbA1c and fasting blood glucose levels. All other characteristics were similar between groups (Table 1).

**TABLE 1:** Donor characteristics at the time of skeletal muscle biopsy collection in severely obese female donors with type 2 diabetes (T2D) and normal glucose tolerance (NGT).

	NGT (n = 6)	T2D (n = 6)
Age (Years)	44.0 ± 7.6	49.2 ± 11.8
BMI (kg/m^2^)	43.3 ± 4.2	45.0 ± 5.4
HbA1c (%)	5.5 ± 0.2	6.7 ± 1.2*
Fasting glucose (mmol/L)	5.0 ± 0.4	6.8 ± 0.9*
Total cholesterol (mmol/L)	4.7 ± 0.8	4.8 ± 1.3
LDL (mmol/L)	2.9 ± 0.6	2.8 ± 1.1
HDL (mmol/L)	1.1 ± 0.2	1.1 ± 0.4
Triglycerides (mmol/L)	1.8 ± 0.6	2.1 ± 0.8

Data is presented as average ± standard deviation. * Indicates t-test p < 0.05.

### 3.2 sEV number, size, and surface markers Do Dot differ between groups

**FIGURE 1:**
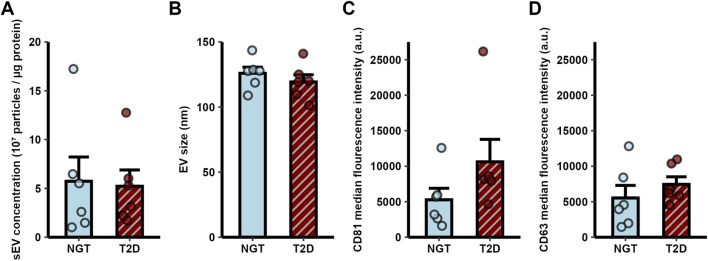
Characterization of small extracellular vesicles (sEVs) derived from human myotubes of severely obese female donors with normal glucose tolerance (NGT, n = 6) and type 2 diabetes (T2D, n = 6). **(A)** sEV concentration and **(B)** diameter were measured by nanoparticle tracking analysis. Flow cytometry analysis of a CD81 positive sEV population confirmed the presence of the EV markers **(C)** CD81 and **(D)** CD63. No significant differences were observed between groups (t-test p > 0.05). Data are presented as mean ± SEM.

Myotube-derived sEVs originating from differentiated skeletal muscle donor cells in culture were characterized by size and concentration using nanoparticle tracking analysis, and by the surface markers CD63 and CD81 using flow cytometry. The mean concentration of sEVs isolated from the NGT group was 5.7 × 10^7^ ± 2.5 × 10^7^ particles/µg cellular protein, while in the T2D group it was 5.2 × 10^7^ ± 1.7 × 10^7^ particles/µg cellular protein (Mean ± SEM). No significant difference was observed between the two groups (Figure 1A). The average size of sEVs was also similar between the groups, with NGT sEVs measuring 125.9 ± 4.7 nm and T2D sEVs measuring 119.3 ± 5.5 nm (Figure 1B, Mean ± SEM).

Flow cytometry analysis confirmed the presence of the surface markers CD81 and CD63 on sEVs. No significant differences in the median fluorescence intensity of these markers were observed between the groups (Figures 1C,D).

### 3.3 Typical EV shape observed by TEM

**FIGURE 2:**
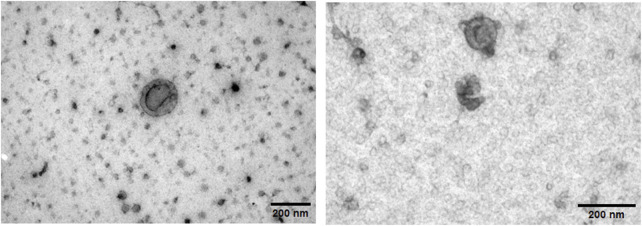
Transmission electron microscopy images of freshly isolated human myotube-derived small extracellular vesicles. Both images are of sEVs from the same T2D donor. Scalebar = 200 nm.

Transmission electron microscopy revealed that the isolated sEVs exhibited the characteristic cup-shaped morphology typically associated with extracellular vesicles when analyzed by TEM, confirming their identity (Figure 2).

### 3.4 Distinct proteomic profiles in sEVs from T2D and NGT donors

**FIGURE 3:**
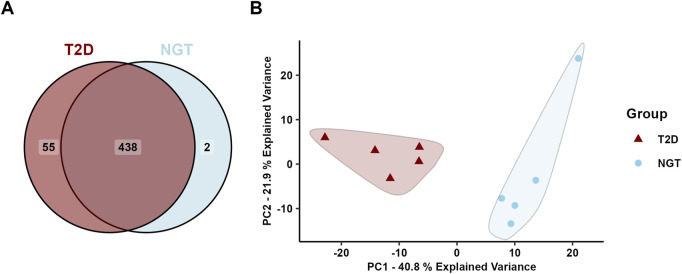
Protein cargo in small extracellular vesicles (sEVs) isolated from human myotubes of severely obese female donors with normal glucose tolerance (NGT, n = 5) and type 2 diabetes (T2D, n = 5). **(A)** Distribution of identified proteins. **(B)** Principal component analysis of all identified proteins revealed two distinct clusters, one corresponding to the NGT group and the other to the T2D group, suggesting group-specific protein profiles.

A total of 495 unique proteins were identified in the sEVs isolates, of which 493 were found in the T2D group and 440 in the NGT group (Figure 3A). Of these, 64 proteins overlapped with the top 100 most frequently identified proteins in EV studies from Vesiclepedia (Chitti et al., 2024), supporting the EV origin of these samples.

Principal component analysis (PCA) of all identified proteins revealed two distinct clusters corresponding to the two groups, indicating a clear difference in the protein cargo of sEVs from these donor populations (Figure 3B). The first principal component accounted for 40.8% of the sample variance, and visual inspection of the PCA plot revealed this to be the main separator of the two group clusters.

To better understand the biological function associated with the identified proteins, pathway analysis was performed (Table 2). The proteins common to both T2D and NGT groups were enriched in canonical pathways linked to EV-associated processes such as granule mobilization and exocytosis. Additionally, among the top metabolic pathways for both groups were *Glycolysis I* and *Gluconeogenesis I*, suggesting the sEV proteins to be involved in glucose metabolism (Supplementary Data).

**TABLE 2:** Top five canonical pathways enriched among all identified proteins (All), proteins unique to the type 2 diabetes (T2D) group (T2D-Unique), proteins significantly differing between groups (Significant), and proteins contributing most to the first principal component (PC1) in the principal component analysis (PCA), which accounted for the largest group separation (PCI). No shared pathways were found for the two proteins unique to the NGT group.

	Rank	Pathway name	-log10(p)	Z-score
**All**	1	Neutrophil degranulation	41.1	3.6
	2	Extracellular matrix organization	39.0	0.3
	3	Response to elevated platelet cytosolic Ca2+	38.9	2.0
	4	Regulation of insulin-like growth factor transport and uptake by IGFBPs	36.0	2.5
	5	Post-translational protein phosphorylation	31.8	2.9
**T2D-unique**	1	Striated muscle contraction	11.7	0.4
	2	Signaling by ROBO receptors	8.7	NaN
	3	Response of EIF2AK4 (GCN2) to amino acid deficiency	8.4	1.1
	4	Eukaryotic translation initiation	7.8	1.1
	5	Dilated cardiomyopathy signaling pathway	7.3	−0.8
**Significant**	1	Neutrophil degranulation	22.1	4.3
	2	Response to elevated platelet cytosolic Ca2+	17.0	3.0
	3	Regulation of insulin-like growth factor transport and uptake by IGFBPs	12.4	2.3
	4	Post-translational protein phosphorylation	12.1	2.1
	5	Collagen degradation	10.9	3.3
**PC1**	1	Response to elevated platelet cytosolic Ca2+	13.4	2.7
	2	Neutrophil degranulation	12.2	4.6
	3	Striated muscle contraction	10.5	2.8
	4	Glycolysis I	10.2	2.5
	5	Gluconeogenesis I	8.3	2.2

P-values and z-scores were generated by Ingenuity Pathway Analysis (IPA), where the p-value reflects the strength of the association. The z-score indicates the predicted direction and magnitude of pathway activity (positive for activation, negative for inhibition), based on differences in protein abundances observed between the groups. NaN = pathway activity prediction unavailable.

The 55 proteins unique to T2D were most significantly linked to skeletal muscle structure and contraction, as well as protein synthesis. Only two proteins were exclusively detected in NGT sEVs, Cathepsin K and Apolipoprotein A1. These shared no pathways, preventing pathway enrichment analysis.

The proteins contributing to the first principal component were most significantly associated with glucose metabolism-related pathways, processes involved in granule mobilization and exocytosis (relevant to EV secretion), and *responses to elevated platelet cytosolic Ca* ^*2+*^ *,* a pathway involving inositol-dependent protein kinase C signaling that overlaps with components of the insulin signaling pathway. All of these processes were predicted to be activated in T2D compared to NGT.

**FIGURE 4:**
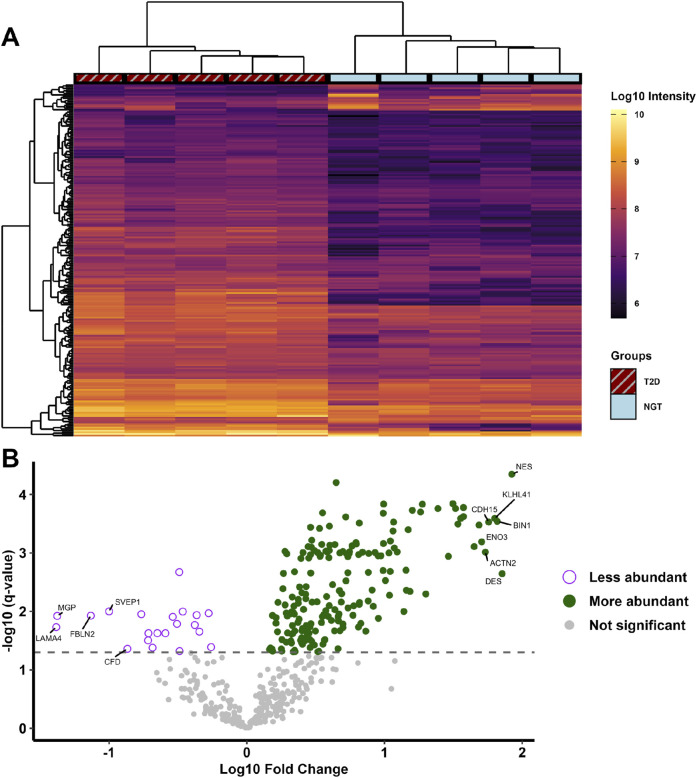
**(A)** Heatmap of proteins significantly different in small extracellular vesicles (sEVs) from myotubes of severely obese female donors with type 2 diabetes (T2D, n = 5) compared to those with normal glucose tolerance (NGT, n = 5). Hierarchical clustering using Euclidean distance was performed to group samples (columns) and proteins (rows) exhibiting similar protein levels. **(B)** Volcano plot displaying all identified proteins, with their fold change values in the T2D group relative to the NGT group, and the corresponding q-values. Significance was determined with unpaired t-tests and permutation-based false discovery rate correction, with the grey dashed line indicates the cutoff q-value of 0.05 (equivalent to -log10(q) value of 1.301). The 7 proteins with the highest and 5 with lowest fold change values are annotated with their respective gene names. (NES = Nestin, DES = Desmin, BIN1 = Myc box-dependent-interacting protein 1, KLHL41 = Kelch-like protein 41, CDH15 = Cadherin-15, ACTN2 = Alpha-actinin-2, ENO3 = Beta-enolase, LAMA4 = Laminin subunit alpha-4, MGP = Matrix Gla protein, FBLN2 = Fibulin-2, SVEP1 = Sushi, von Willebrand factor type A, CFD = Complement factor D).

Comparing the abundance of proteins in the two groups, 194 proteins were found at higher levels and 21 at lower levels in sEVs from T2D group compared to NGT group (Figure 4). Among the proteins with the highest fold changes were the intermediate filament proteins nestin and desmin, Kelch-like protein 41 and alpha-actinin-2 which are involved in sarcomere structure, Cadherin-15 which mediates myoblast adhesion, Myc box-dependent-interacting protein 1 linked to membrane remodeling, and the glycolytic enzyme beta-enolase. The proteins with the lowest fold change in T2D were laminin, matrix Gla protein, fibulin-2, and Sushi von Willebrand factor type A domain-containing protein, all of which are extracellular matrix (ECM) proteins, as well as the immune-related protein complement factor D (Table 3). Among the top pathways for the significantly altered proteins were the EV-related *neutrophil degranulation,* and insulin-signaling associated *response to elevated platelet Ca2+.*

**TABLE 3:** The 15 proteins with the highest and 10 proteins with the lowest fold change values in sEVs from the type 2 diabetes (T2D) group relative to normal glucose tolerance (NGT) group.

Protein names	Gene names	Fold change (relative to NGT)
Nestin	*NES*	83.97
Desmin	*DES*	71.50
Myc box-dependent-interacting protein 1	*BIN1*	65.83
Kelch-like protein 41	*KLHL41*	63.18
Cadherin-15	*CDH15*	57.10
Alpha-actinin-2	*ACTN2*	54.02
Beta-enolase	*ENO3*	50.77
Myosin-7	*MYH7*	48.54
Calsyntenin-2	*CLSTN2*	44.79
Myosin-3	*MYH3*	37.44
Titin	*TTN*	37.43
Creatine kinase M-type	*CKM*	35.76
Tripartite motif-containing protein 72	*TRIM72*	34.13
Calsequestrin-2	*CASQ2*	32.78
Tropomyosin alpha-1 chain	*TPM1*	31.37
Laminin subunit alpha-4	*LAMA4*	0.04
Matrix Gla protein	*MGP*	0.04
Fibulin-2	*FBLN2*	0.07
Sushi, von Willebrand factor type A	*SVEP1*	0.10
Complement factor D	*CFD*	0.14
Inactive serine protease PAMR1	*PAMR1*	0.17
Proteoglycan 4	*PRG4*	0.19
Nidogen-2	*NID2*	0.19
Nidogen-1	*NID1*	0.21
Platelet-derived growth factor receptor beta	*PDGFRB*	0.22

Examining the proteins with increased abundance in the T2D group, the top pathways were *neutrophil degranulation, response to elevated platelet Ca2+, collagen degradation, mitotic G2-G2/M phases,* and collagen biosynthesis and modifying enzymes.

On the other hand, the proteins with lower levels in the T2D group were related to *neutrophil degranulation, post-translational protein phosphorylation, regulation of insulin-like growth factor (IGF) transport and uptake by IGFBPs, response to elevated platelet cytosolic Ca2* ^*+*^, and *interconversion of nucleotide di- and triphosphates.*

### 3.5 miR expression and target pathways in sEVs

**FIGURE 5:**
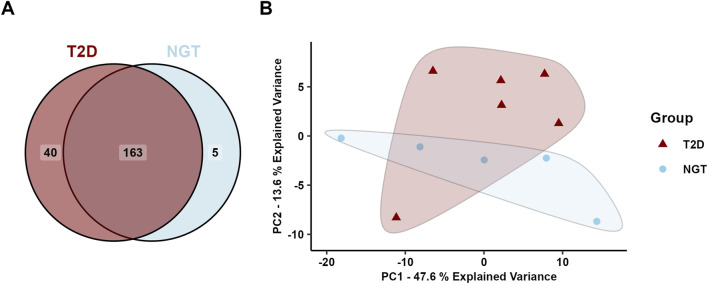
Micro-RNA (miR) content in small extracellular vesicles (sEVs) isolated from human myotubes of severely obese female donors with normal glucose tolerance (NGT, n = 5) and type 2 diabetes (T2D, n = 6). **(A)** Distribution of detected EV miRs in the two groups. **(B)** Principal component analysis (PCA) of all identified miRs reveals considerable overlap of the groups, indicating similar miR content in the two groups.

After filtering the miR data from the arrays, 208 miRs were detected with a satisfactory signal value in at least three samples in at least one group Of these 208 proteins, 40 were uniquely identified in sEVs from the T2D group, 5 were unique to the NGT group, and the remaining 163 were present in both groups (Figure 5A). PCA of the miR data showed overlapping clusters along both the two first principal components, indicating that the miR content in these groups was relatively similar (Figure 5B).

**FIGURE 6:**
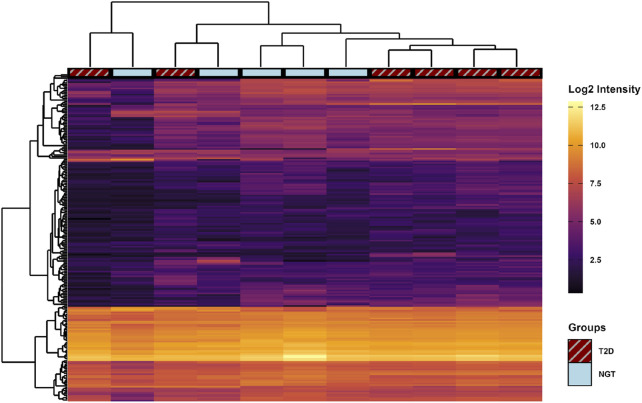
Heatmap of micro-RNA (miR) content profiles in small extracellular vesicles (sEVs) from human myotubes of severely obese female donors with normal glucose tolerance (NGT, n = 5) and type 2 diabetes (T2D, n = 6). Signal intensities of all 208 identified miRs (rows) are shown across individual samples (columns). Hierarchical clustering using Euclidean distance was performed to group similar samples and miRs. No miRs were significantly different between groups following unpaired t-tests with Benjamini–Hochberg correction for multiple comparisons.

Comparing the miR content between the two groups, 6 miRs (miR-5196-5p, miR-551b-5p, miR-23a-3p, miR-663a, miR-6789-5p, and miR-5006-5p) were found at significantly different levels based on raw p-values. However, after adjusting for multiple comparisons by the Benjamini–Hochberg method, no miR differences were found to be significant (Figure 6). Therefore, the 5 miRs with the highest and lowest fold-change values between groups were used for pathway analysis (Table 4).

**TABLE 4:** The 5 miRs with the highest and 5 with the lowest fold change values in the type 2 diabetes (T2D) group relative to normal glucose tolerance (NGT) group.

Top 5		Bottom 5	
miR	Fold change	miR	Fold change
miR-206	9.02	miR-4720-5p	0.39
miR-23a-3p	7.90	miR-3613-5p	0.48
miR-7641	5.81	miR-4330	0.50
miR-1273g-3p	4.96	miR-6808-3p	0.53
Let-7b-5p	4.74	miR-2277-5p	0.59

**FIGURE 7:**
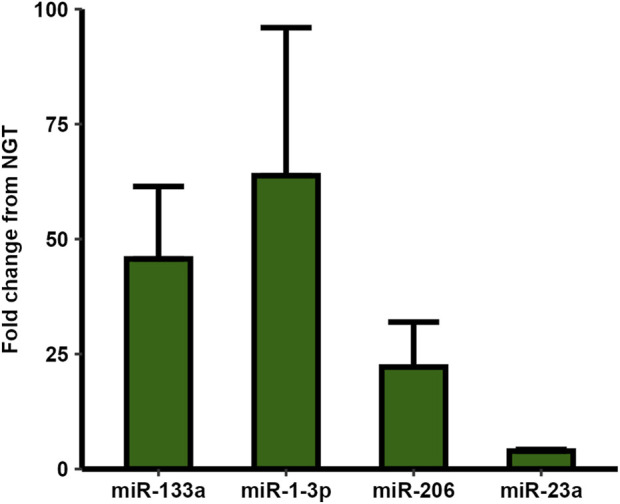
RT-qPCR analysis of selected micro-RNAs (miRs) in small extracellular vesicles (sEVs) from myotubes of severely obese female donors with type 2 diabetes (T2D) compared to those with normal glucose tolerance (NGT). All four examined miRs showed higher levels in the T2D group, though unpaired t-tests revealed no statistically significant differences. Data are presented as mean ± SEM, n = 2-6 per group depending on miR (Donor numbers: miR-1-3p: T2D = 6, NGT = 5, miR-133a: T2D = 6, NGT = 5, miR-206: T2D = 6, NGT = 4, miR-23a: T2D = 2, NGT = 2).

The presence of the known myo-miRs miR-1-3p, and miR-133a was confirmed by RT-qPCR, with average Ct-values of 23.9 ± 3.6. The myo-miR levels did not differ significantly between groups, although both showed a high average fold change in T2D relative to NGT. Additionally, the presence and similar levels of two of the miRs with the highest fold change from the microarrays, miR-23a and miR-206, were also confirmed via qPCR. Again, these miRs showed higher averages in T2D-sEVs without significance (Figure 7).

### 3.6 miR target prediction and pathway analysis

miR target prediction was performed using IPA, with the analysis restricted to experimentally validated or targets predicted with high probability interactions. Across all 208 identified miRs, a total of 45,694 predicted targets were identified, corresponding to 12,350 unique genes. Among these, 1154 encoded proteins were identified in the sEV-proteome, representing 330 unique proteins. Of these miR-protein pairs, 501 (43.3%) were found to have opposite abundance patterns in the T2D group (i.e., when the miR level was higher, the target protein level was lower, or *vice versa*), while 653 (56.6%) showed similar abundance changes in the T2D group relative to the NGT group (i.e., both miR and target protein levels were higher or lower). Pathway analysis of all predicted miR-targets revealed associations with growth- and cell signaling-related pathways (Table 5).

**TABLE 5:** Top five canonical pathways associated with the predicted target genes of all identified miRs (All), miRs unique to the type 2 diabetes (T2D) (T2D-unique) and normal glucose tolerance (NGT) group (NTG-Unique), and the top/bottom 5 miRs based on fold change (Top/Bottom 5).

	Rank	Pathway name	-log10(p)
**All**	1	Molecular mechanisms of cancer	106.0
	2	S100 family signaling pathway	89.0
	3	G-protein coupled receptor signaling	87.5
	4	Cellular effects of Sildenafil (Viagra)	77.4
	5	Cardiac hypertrophy signaling (enhanced)	75.8
**T2D-unique**	1	Pulmonary Healing signaling pathway	9.9
	2	Colorectal cancer Metastasis signaling	9.2
	3	Pulmonary fibrosis idiopathic signaling pathway	9.1
	4	Molecular mechanisms of cancer	9.0
	5	Glioblastoma Multiforme signaling	8.7
**NGT-unique**	1	Caspase activation via Death receptors in the presence of ligand	3.1
	2	WNT/β-catenin signaling	3.0
	3	Apelin liver signaling pathway	2.9
	4	The visual Cycle	2.7
	5	Serine biosynthesis	2.3
**Top/Bottom 5**	1	Generic transcription pathway	8.9
	2	Pulmonary fibrosis idiopathic signaling pathway	8.1
	3	Activin Inhibin signaling pathway	7.8
	4	Transcriptional regulatory Network in Embryonic stem cells	7.7
	5	Hepatic fibrosis/Hepatic Stellate cell activation	6.8

P-v*alues were generated by Ingenuity Pathway Analysis (IPA), where the p-value reflects the strength of the association*.

For the 40 T2D-unique miRs, 5990 total target interactions were identified, of which 4491 were unique genes. Among these, 158 targets overlapped with sEV-proteins, corresponding to 119 unique proteins. Of these pairs, 53 (33.5%) showed opposite abundance patterns, while 105 (66.5%) exhibited abundance patterns in a similar direction. The T2D-specific miR-targets were predominantly associated with pathways involved in tissue repair and remodeling, cancer-related signaling, and inflammatory responses. Notably, several inositol phosphate-related pathways were among the top-ranked metabolic pathways (Supplementary Data).

The 5 miRs uniquely identified in NGT-sEVs were predicted to target 298 genes, of which 293 were unique. Seven of the predicted targets overlapped with the sEV-proteins, all of which were unique. Of these miR-protein pairs, 6 (85.7%) showed opposite abundance patterns, while only 1 (14.3%) were in the same direction. These target genes were enriched in pathways related to apoptosis regulation, developmental signaling, metabolic processes, and sensory functions.

Finally, analysis of the 5 miRs with the highest and 5 with the lowest fold change between the groups identified a total of 2270 predicted targets, corresponding to 2156 unique genes. Among these, 65 predicted targets were present in the sEV-proteome, of which 58 were unique proteins. In these miR-protein interactions, 22 (33.8%) showed opposite abundance patterns, while 43 (66.2%) were similar. These targets were mainly linked to transcription regulation, tissue remodeling, stem cell function, and cell growth signaling. Similar to the T2D group-unique miRs, multiple inositol-phosphate biosynthesis pathways were identified among the top metabolic pathways (Supplementary Data).

## 4 Discussion

EVs have been shown in multiple studies to contribute to the development of insulin resistance (Deng et al., 2009; Kranendonk et al., 2014a; Kranendonk et al., 2014b; Ying et al., 2017; Mleczko et al., 2018; Yu et al., 2018; Dang et al., 2019; Wang et al., 2025). Although fewer studies have looked specifically at skeletal muscle-derived EVs, they too appear to influence insulin sensitivity (Aswad et al., 2014; Jalabert et al., 2016). However, the molecular content of skeletal muscle-derived EVs of T2D patients remains largely unknown. In this study, we aimed to investigate the differences between myotube-derived sEVs from severely obese donors with T2D compared to NGT. No differences were seen in the number or size of the sEVs secreted, nor in the abundance of the EV markers CD63 and CD81. However, the protein content of sEVs differed markedly, with two distinct proteome-profiles identified for the two groups. Most of the proteins were found at higher levels in the T2D group and were involved in pathways related to vesicle secretion, glucose metabolism, and ECM remodeling. On the miR level, however, some miRs were uniquely found in one of the groups while no significant differences were found between the groups. Notably, the targets of T2D-unique and most highly different miRs were related to inositol phosphate metabolism.

### 4.1 T2D does not increase Myotube sEV secretion

No differences in the number of secreted sEVs were observed, as assessed by total particle concentration using nanoparticle tracking analysis. This was supported by comparable levels of the EV-markers CD63 and CD81 measured by flow cytometry. Our findings contrasts with a previous meta-analysis that reported elevated levels of circulating microparticles in individuals with T2D (Li et al., 2016), as well as positive correlations between EV concentration and insulin resistance (Freeman et al., 2018; Kobayashi et al., 2018). Our findings may be explained by our focus on myotube-derived sEVs, whereas previous studies measured total circulating EVs from multiple cell types. Additionally, other factors such as obesity (Amosse et al., 2018), plasma triglycerides (Kobayashi et al., 2018), and inflammation (Matilainen et al., 2024) may play a more prominent role in the elevation of circulating EVs than T2D alone. Our donors were comparable in obesity and plasma triglyceride levels, and myotubes were cultured in standardized media without inflammatory factors, thereby removing these potential indirect T2D-associated drivers of EV secretion.

### 4.2 Distinct sEV Protein profiles differentiate T2D and NGT

#### 4.2.1 Characterizing sEV Protein profiles Distinguishing T2D and NGT

We were able to identify 495 proteins in our sEV samples, of which 55 were unique to the T2D group, 2 unique to the NGT group, and the rest were shared between the groups. Notably, 64 of these proteins overlapped with the top 100 proteins listed in Vesiclepedia (Chitti et al., 2024), supporting the presence of a characteristic EV-specific protein profile. Furthermore, 84% (416) of the proteins identified in this study overlapped with proteins identified in sEVs from our previous study on myotubes derived from the same donors under similar culturing conditions (Aas et al., 2023). Although the previous study had a different aim, this overlap indicates a stable and reproducible myotube sEV protein profile. Interestingly, the most significantly enriched pathway across both groups was *neutrophil degranulation*, a process involving exocytosis and membrane fusion, linking it to the vesicular origin of the proteins.

PCA of the proteomic data revealed two distinct clusters corresponding to the two groups. This separation occurred mainly along the first principal component, which accounted for approximately 41% of the total variance, indicating distinct protein profiles in myotube-derived sEVs from the two groups. Supporting this, 215 significantly different proteins were identified between the groups, with 194 more abundant and 21 less abundant in sEVs derived from T2D myotubes. This clear separation suggests that T2D affects the protein content of skeletal muscle-derived sEVs, possibly reflecting changes in cellular function or vesicle release, especially since other donor characteristics like obesity and gender were similar between groups.

A previous study investigating the plasma-derived EV proteome in individuals with diabetes and prediabetes identified a limited number of proteins with significantly different levels (Alexandru et al., 2022), none of which overlapped with those identified in the present study. This suggests that the clear patterns observed in the present study may reflect a skeletal muscle-specific EV signature that is less detectable in plasma due to the relatively low abundance of skeletal muscle-derived EVs in circulation (Estrada et al., 2022). Compared to the proteomic analysis of skeletal muscle biopsies from lean, obese, and diabetic individuals by Hwang et al. (2009), 100 of the 495 proteins identified in the sEVs of the present study overlapped with the 1,218 proteins detected in skeletal muscle. Six of the proteins that were significantly different in sEVs also differed significantly in skeletal muscle of the previous study. Of these, four proteins with gene names: *PDIA3*, *CCT8*, *CCT4*, and *PSMB3*, all involved in protein folding and degradation, were found at higher levels in the T2D group in both sEVs and skeletal muscle. In contrast, alpha-actinin 2 and desmin were more abundant in T2D sEVs but were reported at lower levels in skeletal muscle from the T2D group in the previous study. This may suggest selective degradation and packaging of structural proteins into sEVs in T2D skeletal muscle cells, although this cannot be confirmed without direct comparison within the same samples.

#### 4.2.2 sEV proteins Responsible for group differences are linked to insulin signaling and metabolic pathways

**FIGURE 8:**
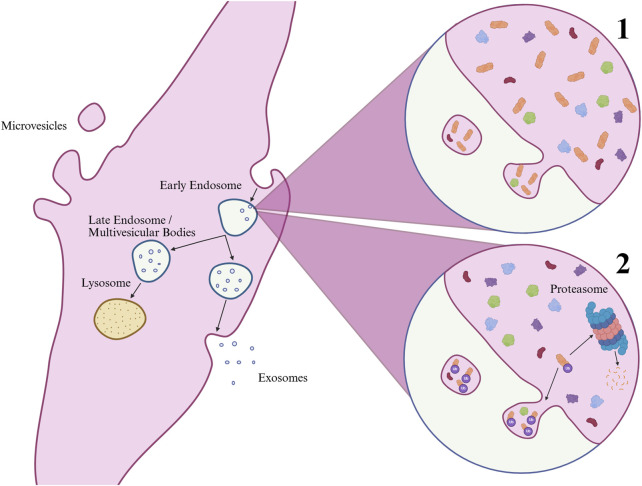
Biogenesis of extracellular vesicles (EVs) and mechanisms regulating exosomal protein content. Microvesicles (typically large EVs) form by outward budding of the plasma membrane, while exosomes (typically small EVs) arise from inward budding of endosomes, generating multivesicular bodies that either degrade in lysosomes or fuse with the plasma membrane for release. Protein enrichment in exosomes may occur through 1) high cellular abundance or 2) post-translational modifications such as ubiquitination, which also targets proteins for proteasomal degradation and may reduce their cellular levels. Created in BioRender. UL, M. (2025) *https://BioRender.com/5pmwq63* .

Pathway analysis of both the proteins contributing to the first principal component and the differentially abundant proteins revealed several pathways associated with T2D. Among the most notable were the *response to elevated platelet cytosolic Ca* ^*2+*^, a pathway involving protein kinase C activation, which has been implicated in disrupting insulin signaling and promoting insulin resistance (Gassaway et al., 2018), and inositol-phosphate signaling, which overlaps with key components of insulin signaling pathways. Chronically elevated Ca^2+^ levels are also found in mice models of T2D and shown to reduce insulin-stimulated glucose uptake (Uryash et al., 2022). Additional associated pathways included *glycolysis I, gluconeogenesis I*, *glucose metabolism*, and *regulation of insulin-like growth factor (IGF) transport and uptake by IGFBPs*. IPA predicts these pathways to be activated, indicating higher levels of the corresponding proteins in T2D-derived sEVs. This can be interpreted in two ways: The proteins could be more abundant in the cells themselves, and therefore more likely to be packed into sEVs. As the main impairment in insulin signaling in T2D is thought to occur at the level of insulin receptor substrates rather than downstream signaling components (Rui et al., 2002; Hançer et al., 2014), compensatory increases in downstream proteins are possible, although several cellular mechanisms likely contribute (Accili et al., 2025). Alternatively, the increased presence of these proteins in sEVs from T2D cells could indicate targeted degradation, as EV-loading of proteins involves ubiquitination, a key process in protein degradation through the ubiquitin-proteasomal system (Lee et al., 2024). As this study did not assess intracellular protein content directly, both interpretations remain possible (Figure 8).

#### 4.2.3 The role of unique proteins in T2D and NGT sEVs

Proteins uniquely detected in T2D-sEVs were associated with pathways involved in skeletal muscle structure and function, such as *striated muscle contraction*, and *dilated cardiomyopathy signaling pathway*. Furthermore, pathways involved in protein translation, including *eukaryotic translation initiation, ribosomal quality control signaling pathway*, *protein folding*, *eukaryotic translation elongation,* and *eukaryotic translation termination* were found. While the translation-related pathways were predicted to be activated, pathways related to skeletal muscle structure showed mixed modulation. This may indicate an increased rate of protein synthesis, enhanced protein turnover or EV packaging, or a combination of these processes in T2D cells. *In vivo* studies have found that protein synthesis rates are generally unaltered in individuals with T2D, whereas protein breakdown is increased and anabolic signaling is impaired (Bell et al., 2006; Cuthbertson et al., 2017).

There were only two proteins unique to the NGT group, Cathepsin K and Apolipoprotein A1. Cathepsin K is a protease involved in apoptosis and fibrosis following injury (Ogasawara et al., 2018), and it has been linked to cardiovascular disease in chronic kidney disease patients, but notably only in the absence of diabetes (Izumi et al., 2016). Apolipoprotein A1, the main component of high-density lipoproteins, has been shown to improve glucose disposal in skeletal muscle cells by activating the insulin signaling pathway (Tang et al., 2019), although its presence in our sEV samples should be interpreted cautiously given its primary synthesis in the liver and intestine.

#### 4.2.4 The top differentially abundant T2D sEV proteins show changes in muscle structure, metabolism, and extracellular matrix remodeling

Among the proteins that were found at higher levels in T2D sEVs, several with established roles in skeletal muscle structure and function were identified. The intermediate filament proteins nestin and desmin, both involved in cytoskeletal organization, showed markedly higher levels in T2D. Proteins related to sarcomere structure, including Kelch-like protein 41 and alpha-actinin-2, were also highly abundant. In addition, Cadherin-15, which mediates myoblast adhesion, and Myc box-dependent-interacting protein 1, linked to membrane remodeling, were also among the top differentially abundant proteins. Notably, the glycolytic enzyme beta-enolase was also found at significantly higher levels.

As mentioned, the protein content of EVs can be influenced both by the cellular abundance of proteins and by selective sorting mechanisms during EV biogenesis involving ubiquitination (Lee et al., 2024). Insulin resistance itself acts as a catabolic signal that promotes ubiquitination and proteolysis, partly through the upregulation of the muscle-specific E3 ubiquitin ligases MAFbx and MuRF1 (Wang et al., 2006). The enrichment of structural proteins could therefore be explained by targeted sorting mechanisms, potentially driven by increased ubiquitination of these proteins in insulin-resistant myotubes. Supporting this notion, structural proteins such as desmin and alpha-actinin have been shown to undergo ubiquitination during skeletal muscle atrophy (Cohen et al., 2009; Cohen et al., 2012). Desmin is phosphorylated and subsequently ubiquitinated in response to GSK3β activity, a kinase inhibited by insulin signaling (Aweida et al., 2018). This suggests that insulin resistance may facilitate the inclusion of cytoskeletal components into EVs by altering posttranslational regulation. This may also explain why desmin and alpha-actinin were found to be decreased in the skeletal muscle of T2D patients in a previous study (Hwang et al., 2009), but increased in the sEVs in our study.

The higher levels of beta-enolase in T2D sEVs suggests a greater reliance on glycolytic metabolisms, although evidence for this happening *in vivo* in diabetes remains inconsistent (Montgomery and Turner, 2015). Furthermore, how these changes relate to alterations within the cell were not assessed in this study.

Among the proteins found at lower levels in T2D were several ECM components, including laminin, matrix Gla protein, fibulin-2, and Sushi von Willebrand factor type A domain-containing protein. This contrasts with the pathology of T2D, where excessive production of ECM protein leading to basal membrane thickening and microvascular complications is a major harmful effect of prolonged hyperglycemia (Cherian et al., 2009). This discrepancy may reflect that important factors driving ECM accumulation *in vivo*, such as hyperglycemia, inflammation, and the contribution of non-muscle cells, are absent or limited in the *in vitro* setting (Bugyei-Twum et al., 2014; Zhao et al., 2022). Additionally, the vesicular content of these proteins may also be affected by the sorting mechanisms mentioned earlier. Taken together, these findings illustrate a complex remodeling of the sEV protein cargo in T2D that may reflect both cellular adaptations and selective vesicle packaging processes.

### 4.3 Comparative analysis of sEV miR content and predicted roles in T2D

#### 4.3.1 Similar miR-profiles were found in T2D and NGT groups

A total of 208 miRs were identified in sEVs in the present study. However, none were significantly differentially abundant between the T2D and NGT groups after adjusting for multiple comparisons. A substantial number of miRs were, however, uniquely detected in sEVs from the T2D group, and a few were unique for the NGT group. PCA showed no clear grouping patterns, suggesting the diabetic condition does not have a major impact on sEV miR content.

#### 4.3.2 Predicted miR targets and functional implications

IPA was used to predict target genes for the sEV-miRs. In total, 12,350 unique target genes were identified for the 208 miRs. Although most of these interactions are predictions, this fits the narrative that miRs can affect multiple gene targets and that multiple miRs can affect the same target gene (Bartel, 2018), and shows the large potential EVs have in intercellular signaling and regulation. However, in the majority of cases (56.6%) the abundance patterns of both the miR and their targets were in similar direction in T2D compared to NGT, opposing the traditional view of miRs role in translational repression. Multiple factors can explain this discrepancy, including differences elsewhere in the transcription-translation-degradation process, or in selective loading of miRs into sEVs.

Next, pathway analysis was performed on the predicted miR-targets. The top canonical pathways across both groups, as well as the combined data, were all heavily involved in cellular signaling. Interestingly, the top metabolic pathways in the T2D group were all inositol-phosphate related, molecules vital for insulin signaling in skeletal muscle, as well as insulin secretion and apoptotic protection in β-cells (Huang et al., 2018). Exogenous inositol treatment has also been shown to improve markers of T2D (Foster et al., 2016; Pintaudi et al., 2016). This pattern was not observed in the NGT group, suggesting sEV-miRs targeting these compounds could have deleterious effects specific to the T2D group. Additionally, the most significantly associated pathways for the miRs with the highest and lowest fold change were related to basal cellular functions such as transcription and translation, as well as fibrosis/ECM remodeling, suggesting important cellular processes could be targeted by these EV-miRs. Importantly, these results are based on predicted miR targets and trends in fold changes rather than statistically significant differences and should therefore be interpreted with caution.

#### 4.3.3 Functional roles of miRs exhibiting the largest differences

Several previous studies have investigated EV-associated miRs in T2D, primarily from serum (Sou et al., 2024). Notably, Kim e*t al.* (2020) reported increased levels of miR-23a-5p in serum EVs from individuals with T2D, whereas we found the complementary strand, miR-23a-3p, in larger quantities in T2D muscle-derived sEVs, although the difference was not statistically significant. The 3p-strand is known to repress translation of the E3 ubiquitin ligases MuRF1 and MAFbx, key components of the ubiquitin-proteasome system, thereby reducing protein degradation and skeletal muscle wasting (Wada et al., 2011). Although cellular levels decrease under atrophic conditions, such as in diabetic models, its EV levels increase, suggesting selective export during catabolic stress (Hudson et al., 2014). Additionally, miR-23a has been shown to protect against renal fibrosis, attenuate the loss of skeletal muscle mass and function, and enhance insulin sensitivity in adipocytes (Lozano-Bartolomé et al., 2018; Zhang et al., 2018). The sEV export of miR-23a from skeletal muscle may therefore contribute to skeletal muscle atrophy while simultaneously mediating protective effects in recipient tissues.

miR-206 showed a 9-fold increase in T2D myotube-derived sEVs, the largest observed difference, supported by RT-qPCR, although this difference was not statistically significant. miR-206, a myo-miR mainly expressed in skeletal muscle, regulates myoblast differentiation (Sjögren et al., 2015), and glycolytic gene expression (Vinod et al., 2016). Knockout of miR-206 has been associated with increased expression of glycolytic genes, improved glucose tolerance, and protection against high-fat diet-induced metabolic impairments (Vinod et al., 2016). Additionally, miR-206 has been reported to reduce hepatic lipid and glucose production and enhance insulin signaling (Wu et al., 2017). High-fat diets have been shown to increase miR-206 expression in pancreatic islets while reducing its expression in skeletal muscle (Vinod et al., 2016), though some studies report increased skeletal muscle expression in T2D (Dahlmans et al., 2017). These divergent findings illustrate the complexity of miR-206s role in T2D pathophysiology and highlight the need for further research to clarify its tissue-specific functions and its potential role in EV-mediated inter-organ communication.

miR-let-7b-5p was also among the most enriched miRs in sEVs derived from T2D cells, but like the others the observed difference was not statistically significant. Overexpression of the let-7 family impairs glucose tolerance in mice, an effect preventable by silencing these genes (Frost and Olson, 2011). miR-let-7b-5p is also enriched in pancreatic cancer cell-derived EVs and can promote lipid accumulation, upregulate FOXO and STAT3, and downregulate IRS1 and GLUT4 in C2C12 skeletal muscle cells, indicating a role in the development of insulin resistance (Wang et al., 2023). EV-delivered miR-let-7b-5p has further been shown to affect cardiomyocytes and promote cardiac remodeling, a key pathological process in heart failure (Zhang et al., 2024). In contrast, some studies report potential benefits. For example, miR-let-7b-5p has shown protective effects against TGF-β1-induced renal fibrosis (Li et al., 2024). In skeletal muscle, its expression is decreased by aerobic training and increased by a high-fat diet, effects mediated by the exercise-responsive regulators PGC1α and PPARδ (Araujo et al., 2020). However, plasma levels of miR-let-7b-5p have been found to be higher in highly trained individuals compared to untrained controls (Li et al., 2020). Higher miR-let-7b-5p levels may therefore contribute to metabolic dysfunction in T2D, though its expression and effects appear tissue- and context-dependent.

miR-7641 and miR-1273g-3p were also among the miRs with the highest fold changes, again without the difference being statistically significant. Neither has previously been investigated in skeletal muscle, however miR-7641 is shown to be decreased in microvascular endothelial cells under high-glucose conditions, where it is linked to increased apoptosis rate and vascular damage (Che et al., 2021), and miR-1273g-3p is found to be increased in retinal pigment epithelial cells in models of T2D, where it is involved in the progression of diabetic retinopathy (Ye et al., 2017).

None of the miRs showing the lowest fold change values were significantly different, and most of them, including miR-2277-5p, miR-6808-3p, miR-4330, and miR-4720-5p, are poorly characterized in skeletal muscle or T2D. While some have been associated with related conditions, such as miR-2277-5p with glucose homeostasis post-bariatric surgery (Lu et al., 2024) and miR-3613-5p with inflammatory myopathies (Franco et al., 2024), their specific roles in this context warrant further investigation.

Although the discussed miRs all exhibited considerable fold changes between the groups, the lack of statistical significance indicates that these findings should be considered exploratory and interpreted with caution. Further studies are required to determine whether these patterns reflect true differences between T2D and NGT sEVs.

### 4.4 Strengths and limitations

A major strength of this study is the use of primary human myotubes to generate sEVs, enabling the specific investigation of skeletal muscle-derived sEVs while preserving physiological relevance. Additionally, the implementation of standardized cell culture conditions, combined with a closely matched control group, ensures that the observed differences between groups reflect the T2D condition itself, rather than confounding factors such as hyperglycemia, obesity, or inflammation.

Despite these strengths, a key limitation is that measurements were performed exclusively on sEVs, without parallel analysis of the source cells. This restricts the interpretation of our findings, as it remains unclear whether changes in sEV content reflect specific cargo sorting mechanisms or simply mirror alterations in cellular abundance. It would be beneficial for future research to include both sEV and cellular measurements to better elucidate the relationship between vesicle content and cellular changes. This assessment could provide valuable information regarding how T2D affects the sEV biogenesis, helping to clarify the mechanisms behind the observed alterations in sEV cargo in T2D.

Another constraint lies with the donors, where individuals from both the T2D and NGT groups were using medication. Such treatments can potentially affect the sEV release and cargo. For example, metformin has been reported to lower circulating levels of platelet-derived EVs (Simeone et al., 2023), while another study found it increased EV secretion and altered protein cargo in EVs from mesenchymal stem cells (Liao et al., 2021). Statins have also been shown to affect multiple aspects of EV biology, including synthesis, cargo, and uptake (reviewed by Sekhavati et al., 2023). The literature on such effects in skeletal muscle derived EVs is however lacking. While the acute effects of these drugs are unlikely to persist *in vitro*, longer-term influences, such as those mediated by epigenetic modifications, cannot be ruled out. However, recruiting donors with severe obesity and type 2 diabetes who are not on any medication is practically unachievable. Similarly, identifying BMI-matched control donors who are entirely medication-free is also challenging.

While the omics approaches in the present study allow for a broad and unbiased assessment of a large number of molecules, they are resource-intensive and therefore come at the cost of a limited sample size. Previous studies in plasma proteomics have shown that sample sizes around six per group have sufficient statistical power to detect differences above 2-fold change (Zhou et al., 2012). Our final sample sizes of five to six donors per group are close to this range, suggesting a valid detection of larger differences. However, smaller but still biologically relevant differences, such as those potentially present in the miR-data, might have been missed. As with all small-scale exploratory studies, a limited sample size reduces generalizability and increases susceptibility to random biological variation. Nevertheless, despite this limitation, we observed significant results, and remain confident that the main findings of our study depict the real differences in sEV content between these groups.

Although a reasonable number of miRs were detected, the overall signal intensity from the microarrays was low. This may have resulted in the loss of less abundant miRs, potentially obscuring relevant findings. However, we believe that the most abundant, and therefore potentially most biologically relevant miRs, were still captured.

A final limitation, in the broader context, is the reductionistic nature of this study, which focuses solely on skeletal muscle cells. While this approach allows detailed insight into sEVs from a key tissue in T2D, the *in vivo* situation is far more complex, with circulating EVs originating from multiple cell types throughout the body. The proportion of circulating EVs derived from muscle is estimated to be low (Estrada et al., 2022), and although concentrations are likely higher locally within the muscle itself (Watanabe et al., 2022), focusing on a single tissue represents a limitation in capturing the full systemic picture. Nevertheless, studying muscle-derived sEVs in isolation provides a controlled environment to investigate tissue-specific mechanisms, which is difficult to achieve in more complex *in vivo* settings, and allows for clearer interpretation of how skeletal muscle contributes to EV-mediated signaling in T2D.

### 4.5 Conclusion

In conclusion, our findings suggest that the size and number of sEVs secreted from myotubes of severely obese female donors with T2D is the same as from NGT. The protein cargo revealed distinct proteome profiles in sEVs from the two groups. The differentially abundant proteins were among others related to glucose metabolism, skeletal muscle structure, and ECM remodeling. Although the miR data did not show statistically significant differences between the T2D and NGT groups, exploratory analysis of miRs with the largest fold changes and their predicted targets suggested potential associations with T2D. These findings provide insight into how sEV content may reflect T2D-related alterations in skeletal muscle and highlight the potential implications of sEV signaling. While further investigation is needed, these findings provide important insight into disease processes in patients with T2D.

## Data Availability

The datasets presented in this study can be found in online repositories. The names of the repository/repositories and accession number(s) can be found below: https://www.ebi.ac.uk/pride/archive/, PXD068287 https://www.ncbi.nlm.nih.gov/geo/, GSE307642.
